# Management of cardiopulmonary bypass in pregnancy: challenges and progress in maternal-fetal protection

**DOI:** 10.3389/fcvm.2025.1637826

**Published:** 2025-09-29

**Authors:** Ziyuan Dong, Lan Luo, Xiaoli Zhuang, Lin Fu, Shuyuan Yi, Kan Wang, Yu Jiang, Xiaofang Yang, Feilong Hei

**Affiliations:** The Extracorporeal Circulation and Mechanical Circulatory Support Department, Beijing Anzhen Hospital, Capital Medical University, Beijing, China

**Keywords:** cardiopulmonary bypass, pregnancy, maternal-fetal protection, cardiac surgery, perfusion strategies, obstetric anesthesia

## Abstract

Maternal heart disease is a leading cause of maternal mortality, and the number of pregnant women requiring cardiac surgery has steadily increased despite advancements in diagnostic and therapeutic modalities. Cardiopulmonary bypass (CPB), while providing surgical support, introduces significant perioperative challenges due to the altered maternal physiology and the unique vulnerability of the fetus, with fetal mortality substantially exceeding maternal mortality. Therefore, maternal and fetal protection during CPB necessitates a comprehensive, multidisciplinary strategy encompassing preoperative planning, intraoperative modification, and postoperative management. Key elements include the optimization of surgical timing, precise CPB management with a focus on maintaining uteroplacental perfusion, minimization of inflammatory and ischemic injury to vital organs, safe anesthetic protocols, judicious pharmacological therapy, appropriate use of extracorporeal life support techniques, and continuous fetal heart rate monitoring. The implementation of these systematic maternal-fetal protective strategies is critical to improving both maternal and fetal outcomes in this high-risk population.

## 1 Introduction

Cardiac disease in pregnancy, whether pre-existing or associated with pregnancy, significantly contributes to maternal morbidity and mortality. Pre-existing conditions include congenital heart diseases, valvular disease, coronary artery disease, aortic disease, chronic hypertension, and cardiomyopathies while cardiac disease associated with pregnancy involves hypertensive disorders of pregnancy and peripartum cardiomyopathy (1, 2). Cardiac disease affects 1%–4% of pregnancies, posing serious risks to both maternal and fetal outcomes (3). Advances in preconception counseling, diagnostics, and surgery have led to a global rise in cardiac interventions during pregnancy and postpartum (4). When medical therapy fails, surgery becomes necessary, despite perioperative challenges from pregnancy-specific physiological changes (5).

Cardiopulmonary bypass (CPB), essential to cardiac surgery, carries notable maternal and fetal risks in pregnancy. Maternal mortality with CPB ranges from 3%–15%, while fetal mortality reaches 16%–35%, and up to 43% in emergencies (1, 6–8). While maternal mortality appears relatively unaffected by the timing of surgery during pregnancy, fetal mortality decreases markedly in the third trimester (10.3%) compared to the first (44.8%) and second trimesters (34.1%) (9). Despite advancements in perioperative anesthetic management, CPB strategies, and fetal monitoring techniques, fetal complications remain a major common (10). The alterations in the hemodynamics of pregnancy, including increased blood volume, cardiac output, and hormonal influences on vascular tone, necessitate a tailored approach to perioperative care. Moreover, CPB-induced changes such as non-pulsatile flow, systemic inflammatory responses, and hemodilution, and hypothermia further complicate maternal-fetal management. Therefore, a comprehensive understanding of the pathophysiological implications of CPB during pregnancy and the implementation of optimized, precise perioperative strategies are essential for improving maternal and fetal outcomes. However, the existing evidence base remains limited and fragmented. Most publications are confined to case reports or experiential summaries, focusing separately on either maternal or fetal outcomes, with few providing an integrated analysis of the impact of CPB during pregnancy in conjunction with pregnancy-specific management strategies. In particular, there is a lack of systematic evaluations that link pathophysiological mechanisms to preventive approaches for maternal-fetal complications. Moreover, no recent review has comprehensively synthesized perioperative CPB management strategies in pregnant patients.

Therefore, this review aims to fill these gaps by systematically delineating the pathophysiological effects of CPB on both mother and fetus, evaluating the latest perioperative protective measures, and highlighting emerging management approaches to optimize maternal and fetal outcomes in this high-risk population.

## 2 Physiological changes in the maternal body during pregnancy

### 2.1 Hemodynamic changes during pregnancy

To meet maternal-fetal metabolic demands, the cardiovascular system undergoes significant adaptations during pregnancy. Cardiac output (CO) increases throughout the entire course of pregnancy (11). CO, determined by heart rate (HR) and stroke volume (SV), is essential for uteroplacental perfusion (12). Early pregnancy CO elevation results from increased SV, while late-stage rise is mainly driven by HR (12). SV rises during early gestation, peaks in mid-pregnancy, then stabilizes or slightly declines (13). HR rises by 10–20 bpm during pregnancy, peaking in late gestation with a 20%–25% overall increase (14, 15). CO rises sharply in early pregnancy and continues to increase through mid-pregnancy (16). In late pregnancy, CO can increase by 45% in healthy singleton pregnancies; in twin pregnancies, it increases by an additional 15%, and the increase in left atrial diameter is significantly greater than in singleton pregnancies (17). Increased sympathetic tone elevates baseline HR, influencing CO, and it also enhances SV through the activation of the renin-angiotensin-aldosterone system (RAAS) (18, 19). Although RAAS activation-induced sodium and water retention increases end-diastolic volume and CO, this increase in CO does not fully offset the reduction in vascular resistance, which explains the possible drop in maternal diastolic blood pressure (20). After 24 weeks of pregnancy, the enlarged uterus exerts greater pressure on the aorta and inferior vena cava, which may lead to a decrease in CO^2^. CO further increases during labor, peaking after delivery. This is attributed to the removal of the placental vascular bed, redistribution of extracellular fluid post-delivery and the release of uterine pressure on the aorta and inferior vena cava, which enhances venous return (2). Comprehending the physiological hemodynamic adaptations of pregnancy is critical for optimizing perioperative management and improving maternal-fetal outcomes.

### 2.2 Changes in systemic vascular resistance and plasma volume due to hormonal changes in pregnancy

Peripheral vasodilation begins in the 5th week of pregnancy (11). Early maternal vasodilation markedly reduces systemic vascular resistance (SVR), which reaches its lowest point in mid-pregnancy and remains stable or slightly increases in late pregnancy (14). These changes are mainly hormone-driven, particularly by estrogen and progesterone. Estrogen may activate G protein-coupled estrogen receptor 1 (GPER-1) to promoting Nitric Oxide (NO) release and enhancing cyclic adenosine monophosphate (cAMP) signaling to induce vasodilation (21). Progesterone's vasodilatory effects may be achieved by inhibiting L-type Ca^2+^ influx and reducing intracellular calcium concentrations (22, 23).

The increase in blood volume begins in the 6th week of pregnancy and peaks between weeks 30 and 41 (24–26). During normal pregnancy, plasma volume increases by an average of more than 1l, rising between 0.2l and 0.6l by mid-pregnancy (25). By late pregnancy, total plasma volume increases by about 50% compared to pre-pregnancy levels (24). Vasodilation in the maternal vasculature causes insufficient vascular filling, activating the RAAS system to increase plasma volume (25). Angiotensin II enhances sodium reabsorption and raises aldosterone levels, leading to increased sodium and water retention, ultimately boosting blood volume (27). Additionally, increased sympathetic activity during pregnancy stimulates β-adrenergic receptor activation which also raises renin and aldosterone levels and further increases in blood volume (18). Oxytocin is also proven to bind with antidiuretic hormone receptors in the kidneys, causing fluid retention (28). Furthermore, the hemodilution resulting from the increased plasma volume reduces glomerular colloid osmotic pressure, increasing the glomerular filtration rate (29). Elevated relaxin levels also aid in increasing renal blood flow and glomerular filtration (20). The increased plasma volume also increases secretion of atrial natriuretic peptide significantly in late pregnancy to promote the excretion of electrolytes and water (30). In summary, due to the complex neurohormonal interactions, blood volume increases under the influence of multiple factors, but it does not rise indefinitely.

### 2.3 Maternal hematologic changes

During pregnancy, changes in key coagulation and fibrinolytic components result in a hypercoagulable state. Certain coagulation factors, plasminogen, and antifibrinolytic agents all rise during pregnancy (31). Moreover, many pregnant women develop physiological anemia. In reality, individual red blood cells' structure and function remain unchanged during pregnancy (29), and total blood volume, plasma volume, and red blood cell mass all increase to improve maternal oxygen-carrying capacity (13). Physiological anemia still occurs due to dilution, as plasma volume increases early in pregnancy, whereas the rise in red blood cell mass is relatively delayed (32). Additionally, throughout pregnancy, the plasma volume increase is more significant than the growth in red blood cell mass, resulting in decreases in hemoglobin concentration, hematocrit, and red blood cell count (12). In conclusion, these physiological characteristics objectively affect placental oxygen supply and clotting function.

### 2.4 Cardiac disease and cardiovascular complications during pregnancy

During normal pregnancy, physiological changes such as decreased systemic vascular resistance, increased cardiac output, elevated heart rate, and expanded blood volume are generally well tolerated by healthy women. However, in the presence of structural heart disease or limited cardiac reserve, these hemodynamic shifts may precipitate decompensation.

Hypertensive disorders of pregnancy (HDP) are common pregnancy-specific cardiovascular complications that significantly alter maternal hemodynamics and increase cardiovascular risk, and thus have implications for CPB management. They affect approximately 10% of pregnancies and contribute to up to 16% of maternal deaths (33, 34). HDP are traditionally classified into three groups: chronic hypertension, gestational hypertension, and preeclampsia (35). These conditions are usually managed medically and/or obstetrically; however, when cardiac surgery requiring CPB is indicated for other reasons, the hemodynamic alterations associated with HDPs such as intravascular volume expansion, increased vascular permeability, and impaired blood pressure regulation can influence management during cardiac surgery (36).

The hemodynamic tolerance of valvular lesions during pregnancy depends strongly on whether the lesion is stenotic or regurgitant. Stenotic lesions, such as mitral or aortic stenosis, are poorly tolerated due to fixed obstruction and limited cardiac output reserve. In the setting of mitral stenosis, pregnancy-associated tachycardia shortens diastole, raising filling pressures and worsening pulmonary congestion, while atrial fibrillation further increases the risk of atrial thrombus and stroke in the hypercoagulable state (37, 38). All stenotic lesions are sensitive to tachycardia, as the shortened diastolic filling period exacerbates pressure gradients and impairs ventricular filling. Aortic stenosis poses distinct hazards in late pregnancy, where physiologic reductions in diastolic blood pressure and increased heart rate can compromise coronary perfusion, leading to hypotension, angina, and syncope (39). In contrast, chronic regurgitant lesions such as mitral or aortic regurgitation are relatively better tolerated in pregnancy, as the physiologic reduction in systemic vascular resistance decreases regurgitant volume and mitigates pressure overload (37).

Pregnancy-associated acute myocardial infarction (PAMI) is rare but increasing, with an incidence of 1.4–2.5 per 10,000 obstetric admissions (2016–2020, U.S. National Inpatient Sample) (40) and ∼4.5–6.2 cases per 100,000 deliveries in contemporary reviews (41). Spontaneous coronary artery dissection (SCAD) is the leading cause, accounting for ∼30%–40% of cases (37.9% in the NIS cohort), followed by atherosclerotic plaque rupture (∼25%–35%), coronary thrombosis with angiographically normal arteries (∼15%–20%), and coronary vasospasm (∼10%–15%), while takotsubo cardiomyopathy and coronary embolism are less common (∼2%–5%) (41). For pregnant patients presenting with STEMI, primary PCI is recommended over thrombolysis when available (41).

Heart failure in pregnancy may arise from structural or functional cardiomyopathies. Women with preexisting HF face a 7.7-fold increased maternal mortality risk and are more prone to pulmonary edema, renal insufficiency, and stroke. The increased circulatory volume during pregnancy may accelerate decompensation (9).

Aortic dissection during pregnancy is exceedingly rare, accounting for only 0.1% of all aortic dissection cases (42). Approximately 50% of women with pregnancy-related aortic dissection had no prior diagnosis of aortic pathology and were only identified after the dissection occurred (43). Most pregnancy-associated dissections occur during gestation (approximately 61%), with 67% being Stanford type A and 33% type B dissection. Pregnancy-related aortic dissection carries a high mortality, with reported maternal and fetal death rates of 23% and 27%, respectively (44). Elevated levels of estrogen and progesterone during pregnancy may inhibit collagen deposition in the aortic wall, compromising vascular structural integrity (45). Increased relaxin levels contribute to aortic dilatation and increased vascular compliance, rendering the aorta more susceptible to hemodynamic stress (46). The hemodynamic alterations of pregnancy, including increased sympathetic tone, cardiac output, and heart rate, substantially elevate mechanical stress on the aortic wall, thereby exacerbating the risk of dissection (47). Pregnant women with underlying connective tissue disorders are particularly vulnerable, as vascular wall integrity is further compromised under the mechanical load of pregnancy (48). Preconception counseling and close perinatal surveillance are essential for managing aortic pathology in pregnancy. Pregnancy is generally contraindicated when the ascending aorta exceeds 5 cm in diameter, or 4.0–4.5 cm in cases of known connective tissue disease (5).

### 2.5 Effects of CPB on mother and fetus

Procedural intervention is needed when medical management of cardiac problems in pregnancy fails or is not possible. When CPB is needed, the implications of cardiac physiology in pregnancy and impact on the fetus should be considered. During normal pregnancy, the mother's physiological state undergoes significant changes. The drastic changes in the internal environment and hemodynamics during CPB may lead to more harmful effects on the pregnant mother. Although the mother is relatively tolerant of these risks, there is still room for improvement and optimization in current CPB strategies for better protection.

The fetus, however, is particularly vulnerable to CPB. Key fetal risks during CPB arise from: (i) mandatory anticoagulation increasing fetal/neonatal hemorrhage risk; (ii) hemodilution including progesterone dilution predisposing to uterine contractions; (iii) impaired uteroplacental perfusion due to non-pulsatile or low-flow states and CPB-related inflammatory/microcirculatory dysfunction; and (iv) hypothermia and rewarming effects that further compromise fetal oxygen delivery (49–56).

Ultimately, CPB may lead to serious adverse outcomes, including fetal death. In cases where the pregnancy has reached the third trimester and the fetus is viable, cesarean delivery prior to cardiac surgery can significantly reduce fetal mortality (57). In some situations, an earlier delivery may be required for maternal indications; while this strategy can reduce procedure-related fetal mortality, it may increase the risk of neonatal morbidity associated with prematurity.

Secondly, dilution of progesterone concentrations, particularly during the rewarming phase after moderate or deep hypothermia, can increase uterine excitability and stimulate contractions (51). During CPB, contact of blood with the artificial circuit activates complement and neutrophils and disrupts the endothelial glycocalyx and junctions, increasing microvascular permeability and interstitial edema; edema increases the diffusion distance for oxygen. Simultaneously, priming-related hemodilution lowers capillary hematocrit and viscosity, which—despite faster RBC velocity—reduces functional capillary density and creates heterogeneous flow. Together these alterations impair both convective oxygen transport and diffusion at the microcirculatory level (52–55). Hawkins and colleagues observed in fetal sheep experiments that, whether under hypothermic (25 °C) or normothermic conditions, CPB with low flow resulted in low fetal oxygen partial pressure and saturation (56). This suggests that uteroplacental oxygen delivery is markedly affected under low-flow perfusion, underscoring the importance of maintaining high perfusion pressure and flow during CPB to ensure adequate placental perfusion. Each uterine contraction leads to autotransfusion, and maternal acidosis may be transmitted to the fetus (2). Furthermore, uterine contractions caused by CPB may induce fetal acidosis (56). In addition, hypothermia has been reported to cause maternal hypotension and is associated with fetal bradycardia, and these hemodynamic alterations may further compromise uteroplacental perfusion and trigger fetal vasoconstriction (49). Hence, due to factors such as surgery, anesthesia, and organ hypoperfusion, the fetus may experience endothelial dysfunction and elevated catecholamine levels, leading to fetal vasoconstriction and increased SVR (8). At this point, the immature fetal myocardium has poor tolerance to increased SVR, leading to reduced CO and further stress responses, ultimately worsening fetal acidosis and possibly resulting in death (8, 58). Since the fetal circulation involves mixed arterial and venous blood, hemoglobin oxygenation is only 65%. However, fetal hemoglobin (HbF) has a markedly higher affinity for oxygen than adult hemoglobin, so saturation percentage alone does not fully reflect fetal oxygen delivery (59). Therefore, any reduction in placental blood flow and fetal oxygen supply can quickly lead to fetal distress, increasing the risk of fetal death after cardiac surgery (8, 51). The maternal and fetal risks of CPB during different stages of pregnancy, along with suggested management strategies, are summarized in Table 1.

**Table 1:** Maternal and fetal risks associated with CPB during different stages of pregnancy, with suggested management strategies [data adapted from (1, 6–10, 52–55)].

Gestational stage	Maternal risks	Fetal risks	Suggested management strategies
First trimester	Similar overall maternal mortality (3%–15%); greater perioperative hemodynamic instability	Highest fetal mortality (∼44.8%); high risk of abortion and malformations	Avoid elective CPB; if unavoidable, avoid hypothermia, maintain high-normal perfusion pressure and hemoglobin, minimize hypoxia and hypotension, use pregnancy-safe drugs
Second trimester	Maternal mortality ∼3%–15%; increased bleeding risk	Fetal mortality ∼34.1%; risk of preterm labor and growth restriction	Maintain placental perfusion pressure (MAP ≥ 70 mmHg), consider higher pump flow, avoid excessive hemodilution, continuous fetal monitoring, tocolytics if indicated
Third trimester	Maternal mortality ∼3%–15%; aortocaval compression, increased cardiac workload	Lowest fetal mortality (∼10.3%); risk of preterm labor, placental abruption	Left lateral tilt to reduce vena cava compression; prepare for emergency caesarean delivery if fetal distress; coordinate with neonatology
Emergency surgery	Higher maternal mortality; increased multi-organ complication risk	Fetal mortality up to 43%	Rapid pre-op stabilization, multidisciplinary decision-making, intensive intra/post-op maternal-fetal monitoring

## 3 Advances in maternal-fetal protection strategies and CPB management

### 3.1 Preoperative risk assessment and selection of surgical timing

For pregnant patients with underlying heart disease requiring cardiac surgery, comprehensive preoperative assessment is essential.

The primary objective is maternal-fetal survival; in non-emergent cases, timing should be individualized via multidisciplinary risk assessment. For the fetus, fetal mortality decreases significantly as the gestational age advances. For any pregnancy fetal mortality decreases significantly as the gestational age advances. The fetal survival rate is less than 50% for births between 20 and 23 weeks, 83% for births between 24 and 27 weeks, and 94% for births between 28 and 31 weeks (60). In contrast, maternal mortality increases with gestational time as mothers with underlying heart failure experience worsening cardiac function (61). For example, the hemodynamic changes during pregnancy may exacerbate transvalvular pressure gradients in patients with aortic or mitral stenosis, further worsening heart failure, angina, pulmonary edema, and acute arrhythmic symptoms (62, 63). Pregnancy leads to the disruption of the elastic layer of blood vessels and hyperplasia of vascular smooth muscle, affecting the structural integrity of the aortic wall (63–65), which greatly increases the risk of dissection or rupture in pregnant women with aortic diseases compared to normal women (66). The ESC's 2018 guidelines for the management of cardiovascular diseases during pregnancy recommend performing cardiac surgery during the 13th to 28th weeks of pregnancy (5). At this time, maternal hemodynamics are more stable, uterine excitability is minimal, compression of the aorta and inferior vena cava is less significant, and fetal organ development is at a more mature stage, making this the safest period (7, 61).

A recent systematic review reported that maternal mortality was not influenced by the timing of cardiac surgery, whereas fetal mortality was significantly lower in the third trimester compared with earlier stages (50). Cesarean delivery before cardiac surgery can eliminate procedure-related fetal death, but neonatal morbidity increases as gestational age at delivery decreases. Thus, if surgery is non-urgent, and maternal status allows, it should be delayed to the third trimester or a cesarean section should be performed first to maximize fetal survival. In emergency situations, such as when a mother with aortic dissection cannot maintain pregnancy to full term, immediate cardiac surgery to save the mother's life is the top priority. For pregnant women beyond 28 weeks, cardiac surgery can be considered at the time of delivery or shortly after if maternal status and if there is adequate hemostasis following delivery (61). If cardiac surgery is performed during pregnancy, the patient's position should be tilted 15° to the left on the operating table or placed in the left lateral decubitus position to reduce compression of the aorta and inferior vena cava, which can obstruct uteroplacental blood flow (67).

### 3.2 Fetal heart monitoring

Fetal circulation largely depends on heart rate, and bradycardia is typically the first observed indicator of fetal distress. During the initiation and maintenance of CPB, monitoring of FHR may reveal transient or persistent fetal bradycardia (68). Potential causes of decreased FHR may include reduced SVR, decreased uterine blood flow, hemodilution, hypothermia, particulate or air embolism, obstruction of venous drainage during inferior vena cava cannulation, prolonged CPB time, or the use of anesthetic agents (68, 69). FHR monitoring can begin after 18 weeks of pregnancy, but factors like fetal size, position, and maternal obesity can influence the accuracy of the measurements (70). Routine monitoring of uterine tension and the use of FHR monitoring are very effective during surgeries that take place during the third trimester (68, 71, 72). Yajima and Masada reported and validated the effectiveness of real-time FHR monitoring through a femoral vein ultrasound catheter; this technique's advantage lies in its ability to be controlled away from the surgical field throughout the procedure, providing accurate real-time FHR monitoring even during fetal movements. If a decrease or absence of fetal heart rate is detected, the flow and pressure of the CPB circuit should be increased if appropriate, maternal arterial oxygen content should be optimized, and FHR monitoring should continue throughout the procedure and postoperatively.

Nevertheless, the clinical utility of FHR monitoring during CPB depends on the specific context. In emergency, life-saving procedures where maternal survival is the priority, attempting to establish continuous fetal monitoring may delay surgery and is not always appropriate. By contrast, when fetal preservation is intended, FHR monitoring should be performed whenever feasible, as observed changes may help guide intraoperative management—for example, transient bradycardia due to inadequate perfusion may respond to increased pump flow. Ultimately, the decision to apply FHR monitoring should be individualized and made in consultation with senior obstetric and cardiac teams.

### 3.3 Intraoperative CPB management

#### 3.3.1 Advantages of pulsatile perfusion

Pulsatile perfusion technology has undergone long-term development since research began last century. With the use of pulsatile perfusion (PP), placental blood flow significantly increases, effectively reducing fetal systemic vascular resistance (SVR), and leading to higher organ perfusion (73, 74). Nonpulsatile perfusion (NP) may increase placental vascular resistance, thus affecting the placental blood supply and oxygenation function (75). Vedrinne found in a controlled experiment that fetal plasma renin and lactate levels significantly increased only during NP CPB, while in PP CPB, there was a notable increase in plasma NO metabolite levels. This further clarifies the benefits of PP: it likely promotes NO production in the fetal/maternal vascular endothelium, reducing activation of the fetal renin-angiotensin system and vasoconstriction, thus increasing perfusion of the fetal placenta and peripheral vessels, enhancing oxygen delivery to the fetus (76). In the NP mode, some have proposed the use of intra-aortic balloon pump with CPB, which physiologically simulates pulsatile blood flow and improves uterine blood perfusion to some extent (77). PP may also promote recovery of FHR. In a case reported by Masada, invasive ultrasound probes in the femoral vein were used to monitor FHR, and a decrease in FHR was observed after clamping the ascending aorta. Under conditions of normothermic high flow and constant maternal mean arterial pressure, FHR was observed to return to baseline after converting from NP to PP during CPB (78). Furthermore, PP can prevent a significant drop in circulating progesterone concentrations, reducing uterine contraction frequency, stabilizing placental blood flow, and helping to prevent the development of acidosis (79, 80). With continuous advancements in CPB equipment and techniques, the flaws in pulsatile perfusion have been significantly reduced. Additionally, damage to blood components has improved, and the likelihood of hemoglobinuria in patients has also decreased. Although pulsatile perfusion still adds some operational complexity in many centers, short-term pulsatile perfusion during CPB interruption is still beneficial for the fetus.

#### 3.3.2 Pregnancy specific management of pump flow and mean arterial pressure during CPB

Because of the decrease in SVR during pregnancy, maximum dilation of placental vessels, and physiological increases in CO, we currently utilize higher pump flow rates to ensure sufficient perfusion pressure in the systemic circulation during CPB. This approach aids in sustaining blood and oxygen supply to both the uterus and placenta. During CPB, a decrease in uterine blood flow and perfusion pressure can initiate a vicious cycle that compromises placental perfusion and gas exchange capabilities. This risk may be exacerbated under low-temperature conditions, further decreasing the efficiency of oxygen exchange in the placenta (78). Since heart surgery performed during CPB usually takes place at temperatures lower than normal body temperature, pump flow rates and mean arterial pressure are crucial for ensuring fetal oxygenation. Therefore, it is recommended to use higher flow rates (2.5–3.5 L·min⁻¹·m⁻^2^) and perfusion pressures (>70 mm Hg) during CPB to maintain adequate uterine blood flow (71, 81, 82).

#### 3.3.3 Temperature management

Hypothermia can improve myocardial protection by reducing metabolic demand and alleviating tissue hypoxia caused by inadequate perfusion, thereby providing additional protection to the body, especially to high-oxygen-demand organs (83). Nonetheless, FHR is directly related to maternal temperature (69). Furthermore, low temperatures during CPB can cause uterine contractions, decreased placental blood flow, and fetal cardiac arrest (2). Multiple studies have suggested that low temperatures during CPB can decrease fetal oxygen consumption, offering some protective effects for the fetus; however, low temperatures can induce uterine vasoconstriction, which raises the risk of fetal hypoxia (66, 84). In a study involving 69 patients, no fetal deaths occurred in pregnant women undergoing normothermic CPB, whereas the fetal mortality rate in those undergoing hypothermic CPB was 24% (85). Although there are instances of fetal survival during hypothermic CPB, the risks posed by low temperatures to the fetus are still considerable. Cardiac surgeries that require cerebral protection are typically done under moderate to deep hypothermic circulatory arrest (MCHA and DHCA). DHCA can have significant adverse effect for both the patient and fetus, and accordingly employing techniques to minimize or avoid DHCA can have maternal and fetal benefit. For example, during aortic dissection procedures in pregnant patients, if circulatory arrest is required to repair the distal ascending aorta or aortic arch, the common practice for cerebral protection is to use antegrade cerebral perfusion while employing MHCA at a temperature of 28 °C to avoid DHCA, as excessive hypothermia may increase fetal mortality (86).

The rewarming process is equally important; rapid rewarming or excessive body temperature may be associated with the occurrence of brain injury (87). Cooling and rewarming during CPB can lead to sustained uterine contractions, resulting in fetal distress. Therefore, the current consensus recommends maintaining normothermic perfusion (>35 °C) during CPB for surgery in pregnant women whenever possible (5, 71). However, under normothermic conditions, the rewarming rate of the left ventricular myocardium may accelerate, complicating myocardial protection. It may be necessary to infuse more cardioplegic solution during surgery to protect the heart, but care must be taken to avoid excessive hemodilution and elevated potassium levels resulting from this (8).

### 3.4 Drug effects on mother and fetus

During pregnancy, significant pharmacokinetic changes occur, including increased volume of distribution, altered protein binding, delayed gast Effect of high- vs. low-dose tranerointestinal transit, and enhanced drug clearance. These factors may lead to greater fluctuations in free drug concentrations, elevated toxicity risk at initial dosing, and reduced efficacy as pregnancy progresses. Therefore, dynamic adjustment of dosage and dosing intervals is essential to ensure drug safety and efficacy (88).

Management and prevention of thromboembolism and hemorrhage is essential for pregnant patients, particularly those with cardiac disease. Pregnancy itself is a hypercoagulable state, and pre-existing cardiac conditions and valve disease may further increase the risk of thrombosis (2). Low-molecular-weight heparin (LMWH) and unfractionated heparin (UFH) are normally used during pregnancy as they do not cross the placenta and do not have teratogenic effects (88, 89). In the highest-risk patients, such as those with mechanical valves, warfarin may be considered after 12 weeks of gestation once organogenesis is complete, and can be transitioned to LMWH or UFH closer to delivery to facilitate neuraxial anesthesia placement and to decrease bleeding complications (88–90). Standard-dose heparin during CPB is considered safe for the fetus (70, 72). Antifibrinolytic therapy can reduce bleeding and transfusion requirements during cardiac surgery and postpartum hemorrhage, and its use is recommended unless contraindications exist (91–93).

Tranexamic acid (TXA) is the most widely used antifibrinolytic in cardiac surgery and is strongly recommended by the 2024 EACTS/EACTAIC patient blood management guidelines to reduce perioperative bleeding and transfusion requirements (92). The OPTIMAL randomized trial confirmed its efficacy, showing that higher-dose regimens further reduced transfusion needs in adult patients (91). In obstetrics, a large individual patient data meta-analysis demonstrated that TXA decreases life-threatening postpartum bleeding without increasing thrombosis, supporting its established safety in postpartum use (94). However, data on prophylactic TXA during CPB in pregnancy are lacking, and dosing strategies remain undefined. Thus, TXA should not be considered contraindicated, but its use during CPB in pregnancy should be individualized, balancing maternal benefit against limited pregnancy-specific evidence.

Hemodilution during CPB may lead to decreased progesterone levels, which has been proposed as a possible factor contributing to uterine contractions (51); however, there is currently no evidence to support targeted interventions based on this mechanism. Experimental studies in fetal and placental models suggest that glucocorticoids such as dexamethasone or betamethasone can acutely reduce placental vascular resistance and improve perfusion (95, 96). Conversely, broader evidence indicates that antenatal glucocorticoid exposure, especially when excessive or repeated, is associated with reduced placental growth, impaired angiogenesis, and lower birth weight (97, 98). These findings suggest that while glucocorticoids may provide short-term circulatory benefits, their use during CPB in pregnancy should be cautious and individualized, given the absence of robust clinical data.

Common vasoactive medications like dopamine, dobutamine, and epinephrine are not teratogenic or fetotoxic, are safe during breastfeeding (99), and can be used to increase maternal arterial pressure (98). Excessive vasoconstriction of placental vessels is a risk when vasopressors are administered (51, 63, 73). β-blockers such as labetalol and metoprolol are commonly preferred during pregnancy to manage hypertension and may provide protective effects against endothelial dysfunction. However, placental transfer can result in neonatal adverse effects. Recent evidence demonstrates an increased risk of neonatal hypoglycemia and a possible increase in bradycardia following *in utero* β-blocker exposure, and postnatal glucose monitoring for at least 24 h could be considered (100). In a large delivery-day cohort, exposed neonates had higher risks of hypoglycemia (4.3% vs. 1.2%) and bradycardia (1.6% vs. 0.5%) compared with unexposed neonates (101). Registry data also associate β-blocker use with small-for-gestational-age (SGA) infants (adjusted OR: 1.7, mean birth-weight −177 g), with agent-specific differences: labetalol carried the lowest SGA risk, whereas atenolol was associated with the highest risk (102). Atenolol should therefore be avoided due to its established association with fetal growth restriction (103, 104), while labetalol and metoprolol remain the preferred options when β-blockade is required. Patients with Marfan syndrome are advised to routinely use β-blockers preoperatively, as they help reduce myocardial contractility and pulse pressure, relieving stress on the aortic wall and improving its elastic properties, thus decreasing the rate of aneurysm expansion (105).

Intravenous anesthetics have become an important component of the anesthesia regimen for CPB cardiac surgeries. Dexmedetomidine (DEX) is a highly selective α2-adrenergic receptor agonist that has been widely used as an adjunct for anesthesia in CPB cardiac surgeries in recent years, helping to alleviate oxidative stress, inflammatory responses, and immunosuppression caused by surgical trauma. DEX administration significantly decreases heart rate prior to CPB while not impacting mean arterial pressure (106). DEX can also protect myocardial contractile and diastolic functions after ischemia-reperfusion injury (107). Furthermore, DEX can suppress the function of the sinoatrial and atrioventricular nodes, reducing the incidence of perioperative tachyarrhythmias (108). A recent randomized controlled trial reported that intravenous dexmedetomidine did not adversely affect neonatal outcomes, supporting its fetal safety (109). Notably, recent evidence further suggests that perioperative DEX use is associated with a reduced incidence of acute kidney injury (AKI) after cardiac surgery, highlighting its potential renal-protective effects (92).

Magnesium sulfate can relieve uterine spasms in pregnant women; however, its use should be approached with caution in women with hypertrophic obstructive cardiomyopathy and valvular disease (99).

### 3.5 Changes to and management of the blood internal environment during CPB

Any factors affecting placental blood circulation during CPB can have significant implications for fetal health. The use of blood products in the mother may cause placental contraction. However, for anemic mothers, transfusing red blood cells helps maintain an appropriate hemoglobin concentration. To mitigate the effects of hemodilution, autologous priming, as well as retrograde or antegrade techniques, are recommended as part of blood conservation strategies (92). Maintaining a hematocrit above 28% during CPB can optimize maternal oxygen transport and secure adequate oxygen delivery to the fetus (57). Hypocapnia may lead to uteroplacental vasoconstriction, while hypercapnia can increase uterine blood flow. Therefore, some early studies suggest that managing α-pH to control CO_2_ levels may be more beneficial for maintaining appropriate carbon dioxide tension and uteroplacental perfusion (57, 78). K+ participates in the endothelial-derived hyperpolarizing factor (EDHF) response in endothelial cells, mediating vasodilation independently of NO and prostaglandins (PG) (110). K^+^ channels in the placenta play a role in promoting arterial vasodilation. By activating K^+^ channels in smooth muscle cells, membrane depolarization occurs, closing voltage-gated Ca^2+^ channels, reducing Ca^2+^ entry, preventing smooth muscle contraction, and ultimately leading to vasodilation. The vasodilatory mechanisms involving K^+^ help maintain normal blood supply to the placenta, which is crucial for fetal oxygenation (109). However, K^+^ can cross the placenta and enter the fetal circulation. Excessive K^+^ can lead to cardiac conduction disturbances in the fetus, potentially resulting in cardiac arrest. Due to the high potassium concentration in cardioplegic solution, it is advisable to aspirate the returning effluent from the coronary sinus to prevent systemic hyperkalemia, while continuously monitoring electrolytes to maintain potassium within normal limits and avoid the adverse effects of excessive K⁺ load. Kopman et al. demonstrated that aspirating cardioplegic effluent from the coronary sinus effectively prevented post-cardioplegia hyperkalemia, maintaining stable serum potassium compared with significant rises observed in controls (111).

### 3.6 Other forms of mechanical circulatory support

The use of other forms of mechanical circulatory support (MCS) in pregnant women is uncommon; however, it can be employed as a life-saving intervention in cases of acute cardiac or respiratory failure during pregnancy, providing robust cardiopulmonary support. IABP demonstrates potential value in perinatal cardiac surgery by increasing blood flow to the distal maternal aorta, particularly to the uterus. Multiple case reports have shown that using the IABP alongside CPB during the perinatal period can physiologically mimic pulsatile flow and enhance uterine perfusion. This approach helps alleviate severe fetal bradycardia and improve fetal outcomes (77, 112, 113).

Extracorporeal membrane oxygenation (ECMO) is now employed not just for severe cardiopulmonary diseases but also for pregnant women experiencing cardiopulmonary complications, aiding them in overcoming critical respiratory or cardiac failure (114). A study by Naoum reviewed extracorporeal life support data from 1974 to 2018, which included 358 pregnant women who received ECMO treatment (114). The results showed a 30-day overall survival rate of 75.4% for mothers after ECMO treatment, a 1-year overall survival rate of 74.3%, a maternal mortality rate of 20%, and a fetal mortality rate of 35%. This indicates that ECMO is an effective bridge for treating severe cardiopulmonary diseases in pregnant women. Although the use of extracorporeal life support technology during pregnancy may increase the risk of maternal and fetal bleeding, complications related to the use of mechanical devices, and systemic anticoagulation risks, current studies indicate that it is effective and relatively safe for treating severe cardiopulmonary diseases in both mothers and fetuses (115–118). A multi-centre cohort study investigated the obstetric management of critically ill pregnant patients who received ECMO support and reported the maternal and fetal outcomes (119). The factors associated with maternal mortality included serum lactate levels (odds ratio per 1 mmol/L increase: 1.21, 95% CI: 1.03–1.41, *P* = 0.02) and respiratory indications for ECMO (odds ratio: 0.21, 95% CI: 0.05–0.95, *P* = 0.04). The most common complications experienced by pregnant patients were hemorrhagic events (119). While research has demonstrated the successful use of ECMO in pregnant women, it is essential to carefully evaluate the health conditions of both the mother and fetus when considering ECMO, as well as to prepare for delivery.

## 4 Discussion

**Figure 1:**
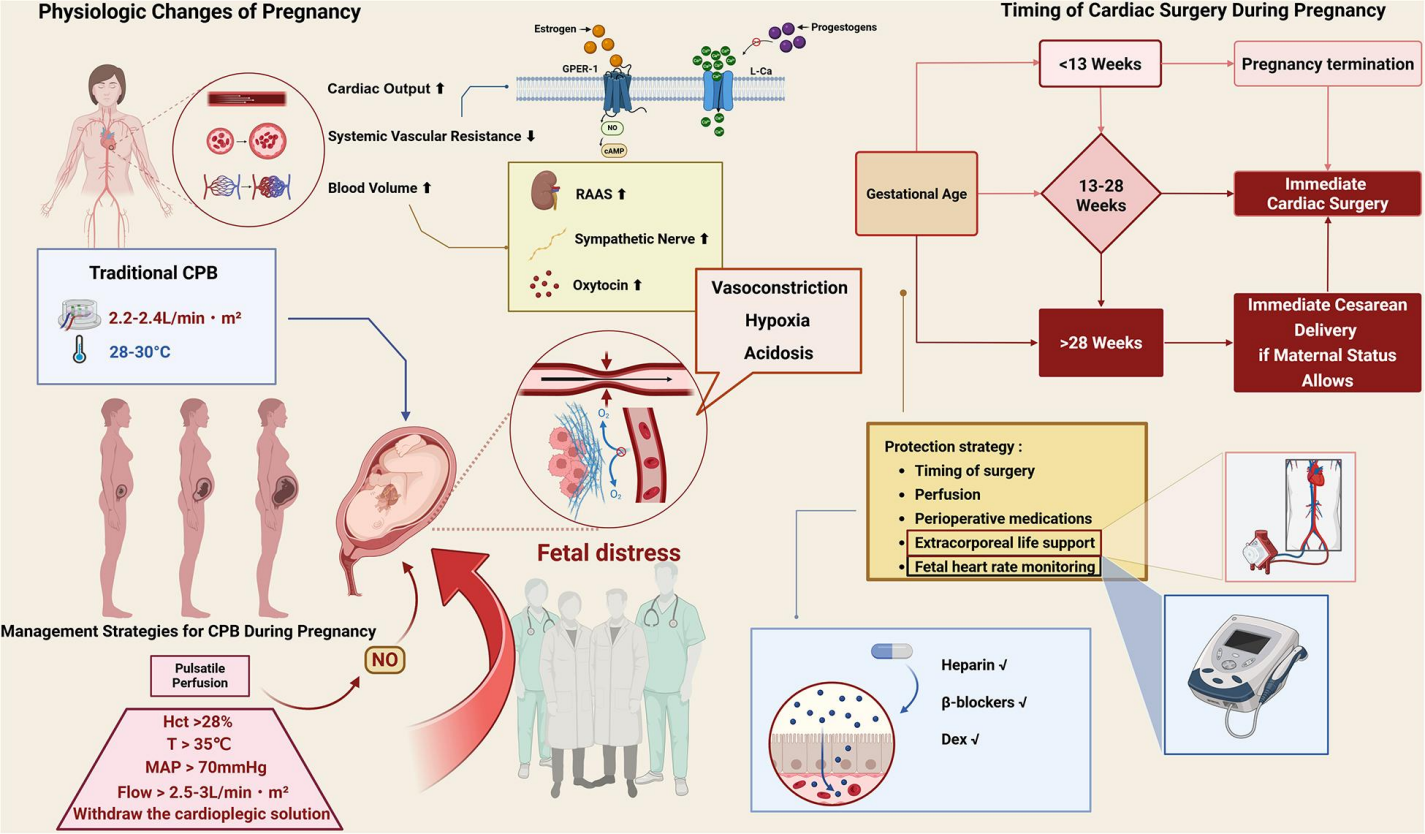
Schematic summary of pregnancy-related physiological changes, CPB risks, and protection strategies. Created in BioRender. Dong, Z. (2025) https://BioRender.com/v11n621. Pregnancy increases cardiac output, blood volume, and systemic vasodilation via hormonal and neurohormonal pathways. Traditional CPB with hypothermia or low flow may lead to vasoconstriction, hypoxia, acidosis, and fetal distress. Protective strategies include maintaining adequate perfusion (flow >2.5–3.0 L/min·m^2^, MAP >70 mmHg, normothermia >35 °C, Hct >28%), timing of surgery, fetal monitoring, and appropriate perioperative medications. Cesarean delivery is recommended before cardiac surgery when gestational age exceeds 28 weeks. GPER-1, G protein-coupled estrogen receptor-1; RAAS, renin–angiotensin–aldosterone system; MAP, mean arterial pressure; Hct, hematocrit; DEX, dexmedetomidine; CO, cardiac output; NO, nitric oxide.

As illustrated in Figure 1, cardiopulmonary bypass during pregnancy requires a multidisciplinary approach for maternal-fetal protection.

In summary, maternal–fetal protection during cardiopulmonary bypass remains a complex clinical challenge characterized by significant difficulties that include maintaining adequate hemodynamics and uteroplacental perfusion, regulating temperature and acid–base balance, and preventing inflammatory and coagulopathic complications while simultaneously ensuring effective fetal monitoring. Despite progress in optimizing CPB management for pregnant women, unresolved issues such as the lack of standardized protocols, limitations in fetal surveillance technology, incomplete understanding of placental responses, and pharmacologic trade-offs necessitate further research (8, 120–122). In addition, it is worth noting that CPB is not always mandatory. In selected cases, alternatives such as off-pump coronary artery bypass (OPCAB) may be feasible, thereby avoiding fetal exposure to CPB and its associated risks.

## 5 Future directions

Future research should focus on developing standardized, evidence-based CPB protocols specifically adapted to the physiological complexities of pregnancy. Particular attention is needed in the following areas:

First, real-time intraoperative fetal monitoring—including uterine artery flow, fetal heart rate variability, and Doppler-based perfusion metrics—should be evaluated for its predictive value in guiding perfusion and anesthetic decisions.

Second, the role of pulsatile perfusion and advanced flow-regulation techniques merits investigation to optimize uteroplacental perfusion during CPB.

Third, strategies for temperature regulation and acid–base management should be refined, particularly considering the dynamic shifts in maternal-fetal thermoregulation and buffering capacity across gestation.

Fourth, the safety and efficacy of pharmacologic agents used during CPB in pregnancy—including anesthetics, vasopressors, and anticoagulants—should be systematically studied, ideally through multicenter registries and translational models.

Lastly, long-term follow-up studies are urgently needed to assess maternal cardiovascular outcomes and fetal neurodevelopment, thereby enabling the establishment of robust clinical guidelines and improving overall survival and quality of life (123, 124).
